# Inhibition of infection-mediated preterm birth by administration of broad spectrum chemokine inhibitor in mice

**DOI:** 10.1111/jcmm.12307

**Published:** 2014-06-04

**Authors:** Oksana Shynlova, Anna Dorogin, Yunqing Li, Stephen Lye

**Affiliations:** a Lunenfeld Tanenbaum Research Institute, Mount Sinai HospitalToronto, ON, Canada; b Department of Obstetrics & Gynecology, University of TorontoToronto, ON, Canada; c Department of Physiology, University of TorontoToronto, ON, Canada

**Keywords:** uterus, infection, preterm labour, leucocyte infiltration, chemokine inhibitor

## Abstract

Preterm birth (PTB) is the single most important cause of perinatal and infant mortality worldwide. Maternal infection can result in PTB. We investigated the ability of a Broad Spectrum Chemokine Inhibitor (BSCI) to prevent infection-induced PTB in mice. PTB was initiated in pregnant mice by intraperitoneal injection of lipopolysaccharide (LPS; 50 μg). Half the mice received BSCI (10 mg/kg) 24 hrs prior to and immediately before LPS administration. The impact of LPS alone or LPS plus BSCI was assessed on (*i*) injection-to-delivery interval, foetal survival rate, placental and neonates' weight; (*ii*) amniotic fluid and maternal plasma cytokine levels (by Luminex assay); foetal and maternal tissue cytokine gene expression levels (by Real-Time RT-PCR); (*iii*) immune cells infiltration into the uterine tissue (by stereological immunohistochemistry). Pre-treatment with BSCI (*i*) decreased LPS-induced PTB (64% *versus* 100%, *P* < 0.05); (*ii*) significantly attenuated cytokine/chemokine expression in maternal tissues (plasma, liver, myometrium, decidua); (*iii*) significantly decreased neutrophil infiltration in the mouse myometrium. BSCI-treated mice in which PTB was delayed till term had live foetuses with normal placental and foetal weight. BSCI represents a promising new class of therapeutics for PTB. In a mouse model of preterm labour, BCSI suppresses systemic inflammation in maternal tissues which resulted in the reduced incidence of LPS-mediated PTB. These data provide support for efforts to target inflammatory responses as a means of preventing PTB.

## Introduction

Preterm birth (PTB) is the single most important cause of perinatal and infant mortality worldwide [1]. PTB is responsible for the majority of newborn morbidity including cerebral palsy, cognitive impairment, blindness, deafness, respiratory illness and complications of neonatal intensive care [2]. Approximately half of PTB is of unknown aetiology, while uterine infection, leading to chorioamnionitis and premature rupture of foetal membranes, is responsible for 30% of all PTB. Systemic maternal infection (*i.e*. pneumonia, pyelonephritis, malaria, typhoid fever, *etc*.) has been associated with preterm labour (PTL) and PTB, although the frequency of these conditions is low in developed countries. Current therapies directed to inhibit myometrial contractile activity have not reduced the incidence of PTB [3].

One factor that appears to be a common element of both infection and idiopathic PTB is the presence of an inflammatory state. There is substantial evidence implying that: (*i*) uterine tissues (decidua and myometrium) express pro-inflammatory cytokines (including chemokines) prior to the onset of term and PTL [4,5]; (*ii*) chemokines from these uterine tissues activate maternal peripheral leukocytes and induce their infiltration into uterine tissue [6]. For instance, macrophage abundance in the human decidua was higher in term labour and non-infection associated PTL than in term non-labouring samples. Neutrophil abundance was unchanged with labour but elevated in PTL with infection [7]. Furthermore, the human myometrium is infiltrated by immune cells during spontaneous non-complicated labour at term [8], as well as labour complicated by uterine infection [9]. Immune cells are themselves a rich source of pro-inflammatory cytokines and prostaglandins [6,10]. As was previously suggested by many researchers, the premature activation of the maternal immune system (either by infection or by other risk factors) can trigger premature myometrial and/or decidual activation (*i.e*. cytokine secretion causing leucocyte influx) and labour onset leading to the delivery of a preterm baby [4,11–13]. Therefore, we hypothesized that these inflammation pathways represent targets for the development of novel therapeutic agents to prevent PTB.

Current data indicate that chemokines and their receptors are involved in the pathological inflammatory reactions of many human diseases, specifically in cancer progression by guiding the migration of tumour cells to secondary organs [14,15] and in the development of HIV (as chemokine receptors are co-receptors for HIV infection) [16]. Pharmacological blockade of chemokine actions by inhibiting their receptors without overall immune suppression has recently been proposed as a novel means of reducing uncontrolled inflammation and preventing disease development [14–16]. Blocking one specific receptor may prevent the actions of multiple chemokines as they induce peripheral leucocyte infiltration into the target tissues. For example, the compound Plerixafor targets CXCR4-CXCL12 chemokine receptors and was used in clinical trial to prevent cancer metastasis [17,18]. Maraviroc (a chemokine CCR5 receptor antagonist) was also found to be effective in early stage HIV therapy [19]. Other studies have used specific chemokine receptor antagonists in rodent models to prevent kidney disease [20–23], bowel inflammation [24,25], stroke and brain damage in the rat [26] or to target tumour growth in the mouse [27]. However, blocking multiple chemokine receptors simultaneously has an advantage of reducing system complexity and enhancing the efficacy of the treatment. Broad Spectrum Chemokine Inhibitor (BSCI, also known as Somatotaxin) has recently been developed to block multiple chemokine signalling pathways simultaneously, while leaving other cytokine signals untouched [28]. BSCI has been shown to exhibit anti-inflammatory activity in a wide range of animal disease models including atherosclerosis [29], surgical adhesion formation [30] and HIV replication [31]. These data have led us to explore (in animal models) whether blocking the signalling of multiple chemokines through the administration of BSCI can reduce uterine inflammation and decrease the rates of PTB. This approach was supported by data showing that a double knockout for interleukin (IL)1 and tumour necrosis factor α (TNF-α) receptor 1 decreased rates of infection-induced PTL in mice while single knockout animals did not [32]. As a proof of principle, we have extensively evaluated the compound (S)-adamantane-1-carbonyl-3-aminocaprolactam called BN83470 (Funxional Therapeutics Ltd, Cambridge, UK) and tested whether this inhibitor can prevent or delay PTB in the mouse model of systemic infection.

## Material and Methods

### Animal model

Hsd:ICR (CD-1) outbred mice used for these experiments were purchased from Harlan Laboratories (http://www.harlan.com/). All mice were housed under specific pathogen-free conditions at the Toronto Centre for Phenogenomics (TCP) on a 12L:12D cycle and were administered food and water *ad libitum*. All animal experiments were approved by the TCP Animal Care Committee. Female mice were mated overnight with males and the day of vaginal plug detection was designated gestational day (GD) 0.5 of pregnancy. The average time of term delivery in our facility was the early morning of GD19. Preterm delivery was defined as the finding of at least one foetus in the cage within 24 or 48 hrs of lipopolysaccharide (LPS) administration. Vaginal bleeding alone was not considered evidence of delivery in the absence of the other signs.

### Experimental design

#### BSCI Administration

**Fig. 1:**
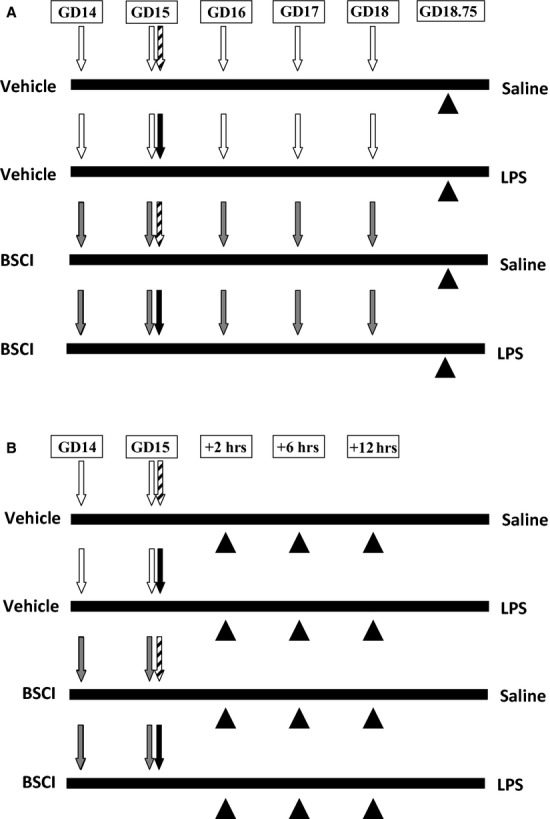
*Scheme of injections and tissue collection*. On GD14, pregnant mice [‘Vehicle’ group (n = 50) and ‘Broad Spectrum Chemokine Inhibitor’ (BSCI) group (n = 50)] were injected subcutaneously with the first dose of BSCI (10 mg/kg/day) or vehicle (ethanol/oil). In 24 hrs, on GD15, pregnant mice received a second injection of BSCI or vehicle. At the same time, half of vehicle group (n = 25) and half of BSCI group (n = 25) received an injection of lipopolysaccharide (LPS; 50 μg). The second half of vehicle group (n = 25) and half of BSCI group (n = 25) received an injection of sterile PBS. The LPS injections were given intraperitoneally. **(A)** Long-term effect of BSCI. Pregnant mice were observed until delivery to record PTD rate. All BSCI-treated animals from LPS and saline groups that did not deliver preterm were given daily injections of BSCI. Mice from both study groups that carry the pregnancy to term were killed before delivery on GD18.75. The number of live pups per litter, the number of foetal resorption sites, birth weights and placental weights were recorded. **(B)** Short-term effect of BSCI. Uterine tissues were collected at 2-, 6-, and 12-hr time-points. Two hours after LPS injection (t = 2 hrs), the first six animals from each groups were killed, and maternal and foetal tissues were collected for analysis. After 4 hrs (t = 6 hrs), six animals from each group were killed and maternal and foetal tissues were collected for analysis. The remaining animals were killed exactly 12 hrs after the administration of LPS (t = 12 hrs) and tissues were collected for biochemical and immunohistochemical evaluation. White arrows represent the day of vehicle injection, grey arrows represent the day of BSCI injection, black arrows represent the time of LPS administration, thatched arrows represent the time of saline administration and black arrowheads represent the time of tissue collection.

Pregnant CD-1 mice (*n* = 100) were randomly divided and one half of animals were subcutaneously (SC) injected with BSCI, the second half received a SC injection of corn oil/ethanol (vehicle). The drug was first administered to pregnant mice on GD14 in the dose that was recommended by the manufacturer, (Funxional Therapeutics Ltd; 10 mg/kg in 100 μl of corn oil/ethanol). Twenty-four hours later, on GD15, mice received the second injection of BSCI prior to the LPS or saline injection (Fig. 1). One daily administration of BSCI at 10 mg/kg ensures that chemokine-induced inflammation remains inhibited continuously for the period of treatment.

#### Systemic infection model

The LPS used for this study was isolated from *Escherichia coli*, serotype 055:B5 (Sigma-Aldrich, St Louis, MO). On GD15, mice received an intraperitoneal (IP) injection of 50 μg of LPS in 100 μl of sterile saline (LPS group, *n* = 50) or IP injections of 100 μl of sterile saline (saline group, *n* = 50). Animals recovered in individual cages and underwent hourly observations using infrared cameras except the interval from midnight to 6 a.m. Starting from 6 a.m., one of the authors (OS or AD) was observing animals every 45–60 min. until delivery. The injection-to-delivery interval was recorded for every animal (Table 1). In two control groups (saline ± BSCI) there were no pregnant mice delivered before term. IP injection of 50 μg LPS/mouse on GD15 causes PTB in 100% animals within 24 hrs with minimal signs of maternal morbidity. All BSCI-treated animals from LPS- and saline-treated groups that did not deliver preterm were given daily injections of BSCI on GD16, GD17 and GD18 (Fig. 1).

**Table 1:** BSCI reduces the incidence of LPS-induced PTB and delays the onset of preterm labour in mice

Treatment	*N*	Preterm delivery rate (%)	Term delivery (%)	Treatment to delivery (h)	Mean N of pups per litter	Term birth weight (g)	Term placental weight (g)
LPS	10	10/10(100)	0	20 ± 4.8	12.3	NA	NA
LPS + BSCI	11	7/11 (64)	4/11 (36)	45.2 ± 26.4*	12.1	0.97 ± 0.15#	0.098 ± 0.007
BSCI	6	0	6/6 (100)	75	14.5	1.3 ± 0.12	0.094 ± 0.015
Vehicle	6	0	6/6(100)	75	13.3	1.1 ± 0.17	0.097 ± 0.018

# Indicates the difference between BSCI group and LPS+BSCI group (*P* < 0.005).

* Indicates the difference between LPS group and LPS+BSCI group (*P* < 0.05).

### Tissue collection

We used different experimental groups to assess short-term and long-term outcome.

#### Long-term outcome study (Fig. 1A)

To evaluate the long-term effect of multiple BSCI treatments, the animals that carried pregnancy to term were killed before delivery from 8 to 10 p.m. on GD18.75 (*n* = 6 in saline group, *n* = 6 in BSCI group, *n* = 4 in LPS+BSCI group). We recorded (*i*) the number of live pups per litter; (*ii*) the number of foetal resorption sites; (*iii*) birth weights of all pups and (*iv*) placental weights. Mice that delivered preterm (*n* = 10 in LPS group and *n* = 7 in LPS+BSCI group) were killed by carbon dioxide inhalation during PTD. The intact uterus of each female mouse was removed and the total number of foetuses, their vital signs, foetal and placental weights was accounted.

#### Short-term outcome study (Fig. 1B)

To evaluate an immediate effect of BSCI on cytokine expression, we collected maternal and foetal tissues at predetermined times (2, 6 and 12 hrs after the LPS injection, *n* = 5–6/group). (1) Maternal blood was obtained by cardiac puncture in a lithium-heparin microtainer (Microvette, Sarstedt, Germany). Plasma was isolated by centrifugation for 5 min. at 2000 × g, and upper phase was collected and frozen in liquid nitrogen until assayed. (2) Maternal liver was collected. (3) Uterus was placed into ice-cold PBS, bisected longitudinally and dissected away from both pups and placentas. Decidua basalis was cut away from the myometrial tissue and pooled from all implantation sites. (4) Myometria from both uterine horns were pooled. The decidua parietalis were carefully removed from the myometrial tissue by mechanical scraping on ice. Foetal tissues: (5) Amniotic fluid was collected from all gestational sacs, centrifuged for 10 min. at 5000 × g; (6) ten placentas were randomly pooled from both uterine horns. All mouse tissues were flash-frozen in liquid nitrogen and stored at −80°C.

### Real-time polymerase chain reaction (PCR) analysis

Total RNA was extracted from the frozen mouse liver, myometria, decidua and placentas using TRIZOL (Gibco BRL, Burlington, ON, Canada) according to manufacturer's instructions (*n* = 5–6/group). RNA samples were column-purified using RNeasy Mini Kit (Qiagen, Mississauga, ON, Canada), and treated with DNase I (Qiagen) to remove genomic DNA contamination. The process was quality-controlled by measuring yield (μg), concentration (μg/μl), and A260:280 ratios *via* spectrometry using Nanodrop ND-1000 and sample integrity using Experion system (Bio-Rad, Mississauga, ON, Canada). cDNA synthesis was performed per manufacturer's protocol (iScript cDNA synthesis kit; Bio-Rad). Quantitative real-time PCR was performed with Luminoct SYBR Green QPCR READYMIX (Sigma-Aldrich), CFX-96 Real Time System C1000 Thermal Cycler (Bio-Rad) and specific pairs of primers (see Table 2). Aliquots (10 ng) of cDNA were used for each PCR reaction run in triplicates. A cycle threshold (Ct) value was recorded for each sample. Each gene was normalized to the expression of three housekeeping genes (*Ppia*, *Tbp*, *Hprt*) by CFX Manager software (version 2.1). Relative gene expression for LPS-treated animals was presented as the average fold change relative to the vehicle sample collected 2 hrs after LPS administration, using the comparative Ct method (see ABI User Bulletin No. 2).

**Table 2:** Real-time PCR primer sequences of a panel of genes involved in inflammatory response and housekeeping genes

Target genes	Primer sequences	GenBank accession #
*IL-1b*	Forward 5′-GGACCCCAAAAGATGAAGGGCTGC-3′	NM_008361
	Reverse 5′-GCTCTTGTTGATGTGCTGCTGCG-3′	
*IL-6*	Forward 5′-CCTCTCTGCAAGAGACTTCC-3′	NM_031168
	Reverse 5′-CTCCGGACTTGTGAAGTAGG-3′	
*IL-12b*	Forward 5′-AACCAGAAAGGTGCGTTCCTC-3′	NM_008352
	Reverse 5′-ATGCCCACTTGCTGCATGA-3′	
*TNF-α*	Forward 5′-ATGGCCCAGACCCTCACACTCA-3′	NM_013693
	Reverse 5′-TGGTGGTTTGCTACGACGTGGG-3′	
*IL-10*	Forward 5′-GCGGCTGAGGCGCTGTCAT-3′	NM_010548
	Reverse 5′-GGCCTTGTAGACACCTTGGTCTTGG-3′	
*Ccl2*	Forward 5′-AGG TGT CCC AAA GAA GCT GTA-3′	NM_011333
	Reverse5′-TCT GGA CCC ATT CCT TCT TG-3′	
*Ccl4*	Forward 5′-AGCCAGCTGTGGTATTCCTGACCA-3′	NM_013652
	Reverse 5′-TCATGTACTCAGTGACCCAGGGCT-3′	
*Cxcl1*	Forward 5′-CCTGCAGACCATGGCTGGGAT-3′	NM_008176
	Reverse 5′-GTGTGGCTATGACTTCGGTTTGGG-3′	
*Cxcl2*	Forward 5′-GTTTGCCTTGACCCTGAAGCCCC-3′	NM_009140
	Reverse 5′-CCAGGTCAGTTAGCCTTGCCTTTGT-3′	
*Csf2*	Forward 5′-TCGAGCAGGGTCTACGGGGC-3′	NM_009969
	Reverse 5′-GTCCGTTTCCGGAGTTGGGGG-3′	
*Hprt*	Forward 5′-CAGTCCCAGCGTCGTGAT-3′	NM_013556.2
	Reverse 5′-CAAGTCTTTCAGTCCTGTCCATAA-3′	
*Ppia*	Forward 5′-CAC CGT GTT CTT CGA CAT CA-3′	NM_008907.1
	Reverse 5′-CCA GTG CTC AGA GCT CGA AAG-3′	
*Tbp*	Forward 5′-TCCCAAGCGATTTGCTGCAGTCATC-3′	NM_013684
	Reverse 5′-ACTCTTGGCTCCTGTGCACACCA-3′	

### Luminex assay

Plasma and amniotic fluid cytokine levels (*n* = 5–6/group) were quantified using Bio-Plex Pro Mouse Cytokine 7-Plex Array kit (Bio-Rad). Multiplex assay was performed on Luminex 200 system and Bio-Plex HTF (Bio-Rad) in accordance with the manufacturer's instructions. Standards and each sample were analysed in duplicate. Data analysis was performed using Bio-Plex Manager, version 5.0 (Bio-Rad) and presented as concentrations (pg/ml).

### Immunohistochemistry analysis

#### Tissue collection

Twelve hours after LPS or vehicle administration, the whole uterus was collected for immunohistochemistry: one intact uterine horn was cut into 10–12 mm segments and placed in 10% neutral buffered formalin (Harleco, Baltimore, MD, USA) or 4% paraformaldehyde (PFA, Electron Microscopy Sciences, Hartfield, PA, USA) for fixation. Samples (*n* = 4/group) were fixed for 24 hrs at 4°C.

#### Immunostaining

Immunostaining was performed as described in [4,5]: The fixed uterine tissues were gradually dehydrated in ethanol and embedded in paraffin. Sections of 5-μm thickness were collected on Superfrost Plus slides (Fisher Scientific, Nepean, ON, Canada). Paraffin sections were deparaffinised and rehydrated. After immersion in 3% hydrogen peroxide (Fisher Scientific, Fair Lawn, NJ, USA), the antigens were unmasked using a microwave heating retrieval treatment in 10 mM sodium citrate solution (pH6) for formalin-fixed tissue and using 0.125% trypsin for PFA-fixed tissue. Blocking was performed for 1 hr with DAKO Protein Serum-Free Blocking solution (DAKO Corporation, Carpinteria, CA, USA). Formalin-fixed tissue was incubated with primary anti-Neu7/4 monoclonal rat antibody (1:100; Cedarlane, Burlington, ON, Canada) overnight. Neu7/4 recognizes the polymorphic 40 kD antigen expressed by polymorphonuclear cells, but absent on resident tissue macrophages. Neu7/4 has low expression on monocytes. PFA-fixed tissue was incubated with primary anti-F4/80 monoclonal rat antibody (1:100; BioLegend, San Diego, CA, USA). F4/80 recognizes the 160 kD glycoprotein expressed majorly by murine macrophages and has low expression on monocytes and eosinophils. For the negative controls, ChromPure non-specific rat IgGs (Jackson Immuno Research Labouratories, West Grove, PA, USA) were used at the same concentration and sections were also incubated with secondary antibodies in the absence of primary antibodies. Detection was accomplished using biotinylated rabbit anti-rat IgG (1:200; Vector Labouratories, Burlingame, CA, USA) in combination with Streptavidin HRP (DAKO Corporation). Final visualization was achieved using Dako Liquid DAB+ Substrate Chromogen System (DAKO Corporation). Counterstaining with Harris' Haematoxylin (Sigma-Aldrich) was carried out before slides were mounted with Cytoseal XYL (Thermo Scientific, Kalamazoo, MI, USA).

### Assessment of leucocyte infiltration using Newcast software

Infiltration of macrophages and neutrophils was quantified using NewCast stereology software with systematic randomized sampling of 2–5% of the total myometrial or decidual area as described in [4,5]. Uterine tissues from LPS-injected group, LPS+BSCI group along with appropriate control groups (*n* = 4/group) were observed on an Olympus BX61 microscope (Markham, ON, Canada) and recorded using an Olympus DP72 camera. The population of leucocytes was assessed for Neu7/4 and F4/80 immunostaining to identify tissue neutrophils/monocytes and macrophages/monocytes respectively. In each uterine tissue sample NewCast Software, part of Visiopharm integrator system 3.6.5.0 (Visiopharm, Hoersholm, Denmark), was used to generate 25 non-contiguous, randomly selected fields of myometrium and of decidua. The number of cells having positive staining for Neu7/4 and F4/80 in each field was counted at 20× magnification and total number of positive cells for 25 fields was normalized for the area of the tissue layer, enabling calculation of the number of positive cells/mm^2^.

### Statistical analysis

Grubbs' outlier test was utilized to identify and exclude outliers from all data sets. To determine differences between cytokine gene and protein expression study groups were subjected to a two-way anova followed by Newman–Keuls posttest (for normally distributed data) or to Kruskal–Wallis non-parametric test followed by Dunn's posttest (for not normally distributed data). PTB outcome and the leucocyte infiltration data were analysed by one-way anova. Normality test and equal variance test were performed by Sigma Stat statistical program and where required, the data were transformed by an appropriate method (usually natural logarithm function) to obtain a normal distribution. The choice as to whether anova or Kruskal–Wallis test was used is based upon the results of the test for statistical normality. The rank method, Kruskal–Wallis test, was used when the data and the transformed data both reject the hypothesis of normality. Statistical analysis was carried out using GraphPad Prism (version 4) for cytokine expression data or Sigma Stat (version 3.11) for the PTB outcome and the leucocyte infiltration data. The level of significance was set at *P* < 0.05.

## Results

### BSCI reduces the incidence of LPS-induced PTB and delays the onset of PTL

Using the pregnant mouse as a model, we tested whether BSCI can prevent or delay PTB induced systemically by IP injection of LPS. There was no delivery for the first 12 hrs after the administration of LPS, however 100% of animals (*n* = 10) delivered within 24 hrs with an ‘injection-to-delivery interval’ of 20.1 + 4.8 hrs (Table 1). We did not detect any maternal mortality and only minimal morbidity. Pre-treatment of pregnant mice with BSCI (10 mg/kg/day) significantly reduced the incidence of LPS-mediated PTB (from 100% to 64%, *P* < 0.05) and delayed the onset of PTB (from 20.1 ± 4.8 to 45.2 ± 26.4 hrs, *P* < 0.05). After administration of LPS and/or BSCI, the mean number of live pups per litter was not significantly different from saline group (Table 1). In addition, we compared the foetal and placental weight of term foetuses that received daily injections of BSCI (*n* = 6) starting from GD14 till term and foetuses injected with saline (vehicle, *n* = 6) or BSCI+LPS. We could not compare the foetal and placental weights between term vehicle-treated or BSCI-treated mice and the LPS-injected group because all animals injected with LPS delivered preterm. Importantly, foetuses injected with LPS in which PTB was delayed till term with the help of BSCI (LPS+BSCI group) were alive and had normal placental and foetal weight (Table 1). Interestingly, the foetal weights of the term BSCI-treated pups (six mice, 87 pups) was significantly higher (*P* < 0.005) than of foetuses from BSCI-treated mice induced with LPS that carry the pregnancy till term (four mice, 44 pups).

### BSCI attenuates LPS-induced cytokine expression in maternal but not foetal tissues

**Fig. 2:**
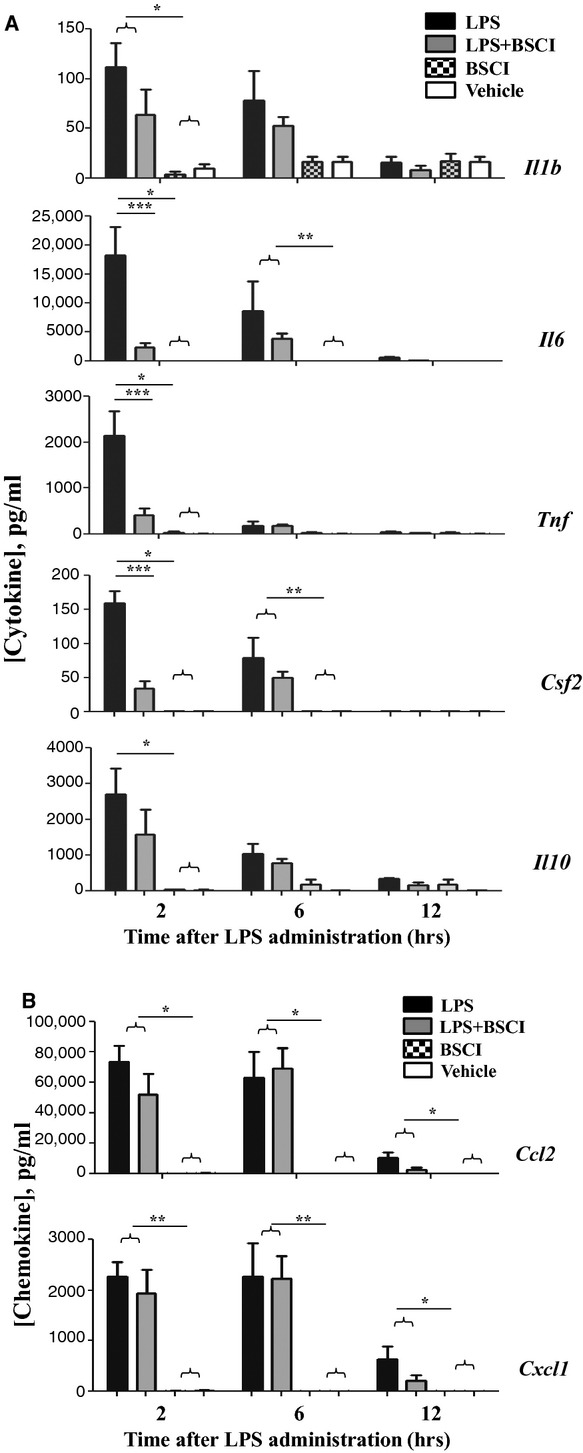
Temporal changes in cytokine protein levels of the maternal blood plasma from GD15 pregnant mice challenged with lipopolysaccharide (LPS) and treated with Broad Spectrum Chemokine Inhibitor (BSCI). (**A**) Pro-inflammatory (IL-1b, IL-6, IL-12 (p40), TNF-α), Csf2 (Gm-scf) and anti-inflammatory (IL-10) cytokines; (**B**) Chemokines Ccl2 (Mcp1) and Cxcl1 (KC/Groa) protein expression were detected by multiplex magnetic bead assay following systemic LPS administration and treatment with BSCI. Shown are vehicle (white bars), BSCI-treated (thatched bars), LPS-injected (black bars) and LPS-injected BSCI-treated samples (grey bars), *n* = 5–6/group. Two-way anova was utilized followed by Bonferroni posttest. Results were expressed as mean ± SEM. Significant difference with vehicle is indicated by * (*P* < 0.05), ** (*P* < 0.01) and *** (*P* < 0.001).

To investigate the direct effect of BSCI on LPS-mediated pro-inflammatory cytokines and chemokines, we measured mRNA expression and protein production in maternal (liver, myometrium, decidua, plasma) and foetal (placenta and amniotic fluid) tissues. Firstly, we determined the effect of BSCI on the immune response to LPS in maternal plasma 2, 6 and 12 hrs after the induction of inflammation. We used the Luminex protein assay which allowed simultaneous measurement of multiple cytokines: IL-1β, IL-6, IL-10, TNF-α, granulocyte-macrophage colony-stimulating factor (Gm-csf/Csf2) and chemokines (C-X-C ligand 1 (Cxcl1, also known as KC) and C-C ligand 2 (Ccl2, also known as Mcp-1). As was expected, within 2-hr systemic administration of LPS dramatically increased plasma levels of all cytokines and chemokines assayed as compared to the plasma levels in the saline-injected animals (Fig. 2, *n* = 5–6). All cytokine proteins were decreased after 6 hrs and further reduced to control levels by 12-hr time-point. Remarkably, BSCI significantly (*P* < 0.001) attenuated TNF-α, IL-6 and Csf2 protein levels measured 2 hrs after LPS injection; IL-1β level was also decreased, however non-significantly (*P* = 0.07, Fig. 2A). The chemokine protein levels showed differential regulation: Ccl2 (major chemoattractant for monocytes) and Cxcl1 (major chemoattractant for neutrophils) were significantly elevated in plasma 2–6 hrs after the LPS injection; by 12-hr time-point, chemokine expression was decreased, but remained significantly elevated compared to control animals. In contrast to the effect on cytokines, BSCI did not significantly reduce LPS-induced plasma chemokine levels (Fig. 2). BSCI treatment itself did not affect cytokine or chemokine levels in the control saline-treated mice.

**Fig. 3:**
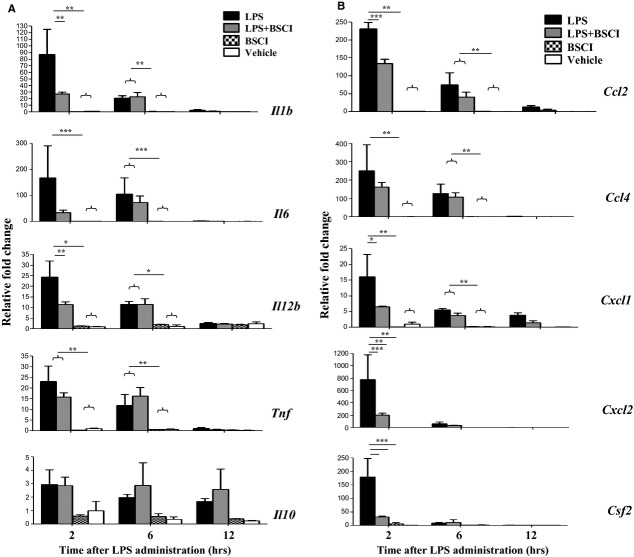
Changes in cytokine mRNA levels in the mouse liver of GD15 pregnant mice following systemic lipopolysaccharide (LPS) administration and treatment with Broad Spectrum Chemokine Inhibitor (BSCI). (**A**) Pro-inflammatory (*IL-1b*, *IL-6*, *IL-12b*, *TNF*, *Csf2/Gmscf*) and anti-inflammatory (*IL-10*) cytokines; (**B**) Chemokines *Ccl2* (*Mcp1*), *Ccl4* (*Mip1b*), *Cxcl1* (*KC or Groa*) and *Cxcl2* (*Mip2a*) mRNA expression were detected by Real-Time RT-PCR. Shown are vehicle (white bars), BSCI-treated (thatched bars), LPS-injected (black bars) and LPS-injected BSCI-treated samples (grey bars), *n* = 5–6/group. Two-way anova was utilized followed by Bonferroni posttests. Results were expressed as mean ± SEM. Significant difference with vehicle is indicated by * (*P* < 0.05), ** (*P* < 0.01) and *** (*P* < 0.001).

**Fig. 4:**
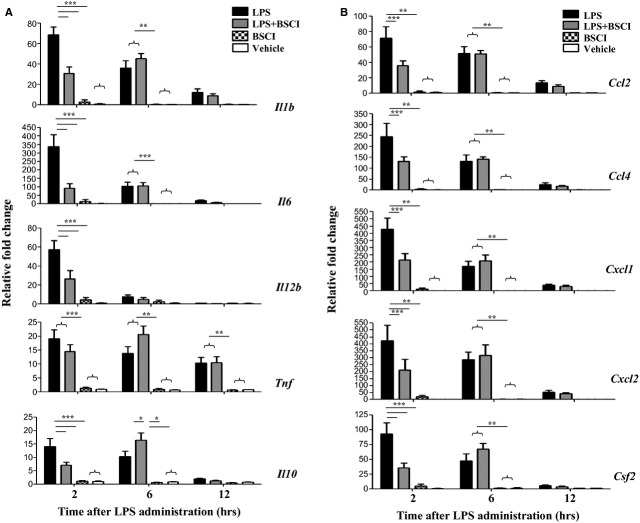
Changes in cytokine mRNA levels in the mouse myometrium of GD15 pregnant mice following systemic lipopolysaccharide (LPS) administration and treatment with Broad Spectrum Chemokine Inhibitor (BSCI). (**A**) Pro-inflammatory (*IL-1b*, *IL-6*, *IL-12b*, *TNF*, *Csf2*) and anti-inflammatory (*IL-10*) cytokines; (**B**) Chemokines *Ccl2*, *Ccl4*, *Cxcl1* and *Cxcl2* mRNA expression were detected by Real-Time RT-PCR. Shown are vehicle (white bars), BSCI-treated (thatched bars), LPS-injected (black bars) and LPS-injected BSCI-treated samples (grey bars), *n* = 5–6/group. Two-way anova was utilized followed by Bonferroni posttest. Results were expressed as mean ± SEM. Significant difference with vehicle is indicated by * (*P* < 0.05), ** (*P* < 0.01) and *** (*P* < 0.001).

**Fig. 5:**
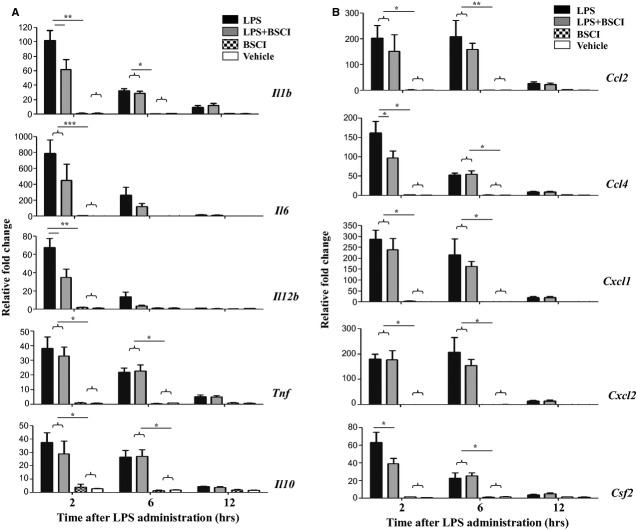
Changes in cytokine mRNA levels in the mouse decidua of GD15 pregnant mice following systemic lipopolysaccharide (LPS) administration and treatment with Broad Spectrum Chemokine Inhibitor (BSCI). (**A**) Pro-inflammatory (*IL-1b*, *IL-6*, *IL-12b*, *TNF*, *Csf2*) and anti-inflammatory (*IL-10*) cytokines; (**B**) Chemokines *Ccl2*, *Ccl4*, *Cxcl1* and *Cxcl2* mRNA expression were detected by Real-Time RT-PCR. Shown are vehicle (white bars), BSCI-treated (thatched bars), LPS-injected (black bars) and LPS-injected BSCI-treated samples (grey bars), *n* = 5–6/group. Two-way anova was utilized followed by Bonferroni posttest. Results were expressed as mean ± SEM. Significant difference with vehicle is indicated by * (*P* < 0.05), ** (*P* < 0.01) and *** (*P* < 0.001).

The expression of multiple cytokine (*IL-1b*, *IL-6*, *IL-12*, *TNF-α*, *Csf2*) and chemokine (*Ccl2*, *Ccl4/Mip1b*, *Cxcl1* and *Cxcl2/Mip2a*) genes was studied in myometrial, decidual and liver samples collected 2, 6 and 12 hrs after the systemic LPS administration (*n* = 5–6/group). All inflammatory genes studied were dramatically up-regulated in the maternal liver 2 hrs after LPS injection (23- to 730-fold increase, Fig. 3) and in reproductive tissues, myometrium (20- to 420-fold increase, Fig. 4) and the decidua (38- to 780-fold increase, Fig. 5) as compared to the tissues from saline-treated animals. Six hours after LPS administration pro-inflammatory cytokine, but not chemokine mRNA levels were decreased in reproductive tissues. BSCI treatment did not affect cytokine or chemokine mRNA levels in maternal tissues of control vehicle-treated mice, however significantly attenuated LPS-induced expression at the 2-hr time-point. All inflammatory genes were down-regulated in the maternal liver, however only changes in *IL-1b*, *IL-12*, *Csf2*, *Ccl2*, *Ccl4*, *Cxcl1* and *Cxcl2* were significant because of the high variability in the expression levels of *IL-6*, *TNF-α*, *Ccl4* (Fig. 3). We detected significant inhibition by BSCI of LPS-induced *IL-1b*, *IL-6*, *IL-12*, *Csf2*, *Ccl2*, *Ccl4*, *Cxcl1* and *Cxcl2* genes in the mouse myometrium (Fig. 4) and *IL-1b*, *IL-12*, *Csf2* and *Ccl4* genes in the mouse decidua (Fig. 5) as compared to LPS-challenged group.

**Fig. 6:**
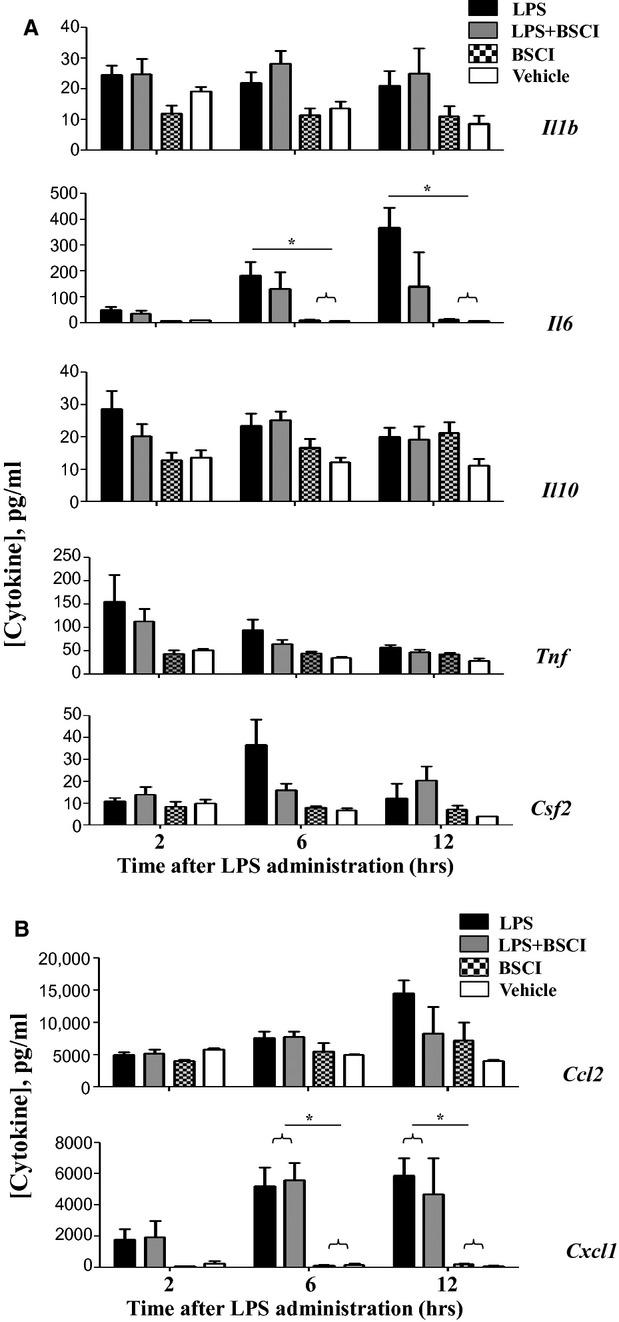
Temporal change in cytokine protein levels of the amniotic fluid from GD15 pregnant mice. (**A**) Pro-inflammatory (IL-1b, IL-6, IL-12(p40), TNF-α, Csf2) and anti-inflammatory (IL-10) cytokines; (**B**) Chemokines Ccl2 and Cxcl1 protein expression were detected by multiplex magnetic bead assay following systemic lipopolysaccharide (LPS) administration and treatment with Broad Spectrum Chemokine Inhibitor (BSCI). Shown are vehicle (white bars), BSCI-treated (thatched bars), LPS-injected (black bars) and LPS-injected BSCI-treated samples (grey bars), *n* = 5–6/group. Two-way anova was utilized followed by Bonferroni posttests. Results were expressed as mean ± SEM. Significant difference with vehicle is indicated by * (*P* < 0.05), ** (*P* < 0.01) and *** (*P* < 0.001).

Importantly, foetal tissues were affected to a less extent by the systemic infection. Only IL-6, Cxcl1 and Ccl2 protein levels were up-regulated in amniotic fluid 6–12 hrs after LPS injection as compared to the saline control, whereas IL-1β, TNF-α and Csf2 proteins were not affected (Fig. 6). BSCI did not affect amniotic fluid cytokines or chemokines levels. Cytokine (*IL-1b*, *IL-6*, *IL-12*, *TNF-α*, *Csf2*) and chemokine (*Ccl2*, *Ccl4*, *Cxcl1* and *Cxcl2*) mRNA levels were up-regulated in the mouse placenta following the systemic LPS administration, however this increase was modest as compared to the immune response recorded in maternal tissues (4- to 42-fold increases, Fig. 8). Importantly, the majority of placental cytokines and chemokines were still highly up-regulated 12 hrs after LPS administration in contrast with the immune reaction of the maternal tissues, whereas cytokine levels returned to control levels at that time. Treatment with BSCI decreased the placental expression of three genes responsible for promoting the infiltration of neutrophils (*Cxcl1*, *Cxcl2* and *Csf2*), however only *Cxcl2* gene expression was reduced significantly (Fig. 8).

**Fig. 7:**
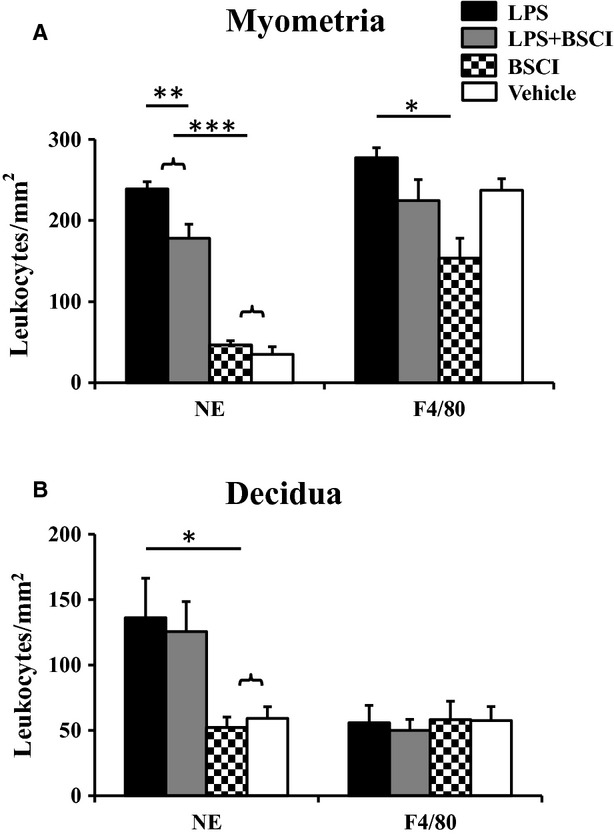
Neutrophil and macrophage infiltration into the mouse uterus 12 hrs after lipopolysaccharide (LPS) and/or Broad Spectrum Chemokine Inhibitor (BSCI) administration. Neutrophils were identified using anti-Neu7/4 antibody and macrophages were identified using anti-F4/80 antibody. NewCast software was used to quantify neutrophil and macrophage numbers in the myometrium and decidua. Shown are vehicle (white bars), BSCI-treated (thatched bars), LPS-challenged (black bars) and LPS-challenged, BSCI-treated (grey bars) samples. Results were expressed as mean ± SEM (*n* = 4). One-way anova was utilized followed by Newman-Keuls posttest. Significant difference with vehicle is indicated by * (*P* < 0.05), ** (*P* < 0.01) and *** (*P* < 0.001).

**Fig. 8:**
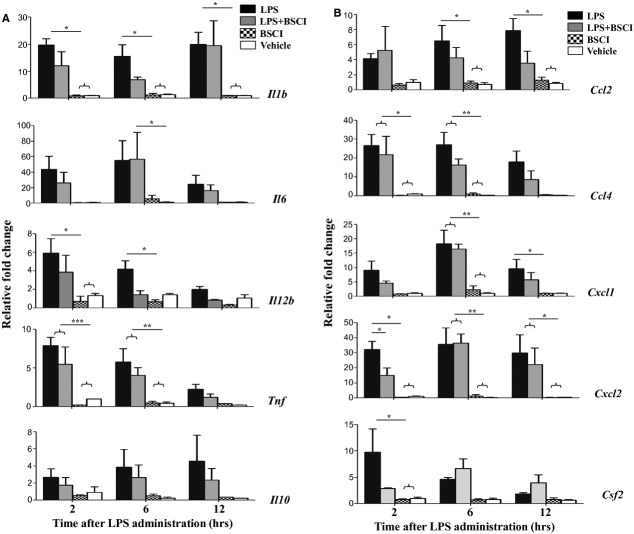
Changes in cytokine mRNA levels in the mouse placenta of GD15 pregnant mice following systemic lipopolysaccharide (LPS) administration and treatment with Broad Spectrum Chemokine Inhibitor (BSCI). (**A**) Pro-inflammatory (*IL-1b*, *IL-6*, *IL-12b*, *TNF*, *Csf2*) and anti-inflammatory (*IL-10*) cytokines; (**B**) Chemokines *Ccl2*, *Ccl4*, *Cxcl1* and *Cxcl2* mRNA expression were detected by Real-Time RT-PCR. Shown are vehicle (white bars), BSCI-treated (thatched bars), LPS-injected (black bars) and LPS-injected BSCI-treated samples (grey bars), *n* = 5–6/group. Two-way anova was utilized followed by Bonferroni posttests. Results were expressed as mean ± SEM. Significant difference with vehicle is indicated by * (*P* < 0.05), ** (*P* < 0.01) and *** (*P* < 0.001).

Anti-inflammatory cytokine IL-10 plays an important role in the suppression of infection-induced inflammatory events because of its pleiotropic effects in immunoregulation. Our data indicate that IL-10 protein expression (1) was highly up-regulated in the maternal blood 2 hrs after systemic LPS administration, reaching 2700 pg/ml levels, which represents a 96-fold increase as compared to the control levels in the vehicle-treated animals (28 pg/ml, Fig. 2A) but (2) was not affected in the AF (Fig. 6A). *IL-10* mRNA levels were slightly, but not significantly up-regulated by LPS injection in the maternal liver (Fig. 3A), not affected in the placenta (Fig. 8A), however significantly increased in the myometrium (13-fold increase, Fig. 4A) and decidua (37-fold increase, Fig. 5A) at 2 hrs time-point. BSCI administration did not affect IL-10 protein and transcript levels in any tissues, except for its down-regulation of the mRNA levels in the myometrium of LPS-injected animals.

### BSCI prevents LPS-induced leucocyte infiltration in the mouse myometrium

To test whether BSCI prevents the LPS-induced recruitment of immune cells into the pregnant uterus, we quantified the infiltration of macrophages and neutrophils into the mouse myometrium and decidua 12 hrs after LPS administration (*n* = 4/group). Visiopharm Integrated System in combination with NewCast stereology software was used to quantify neutrophils and macrophages immunostained *in situ*. Overall, the number of F4/80^+^ macrophages in the mouse myometrium collected from the vehicle group on GD15 was 6-times higher than neutrophils (defined as cells stained positively for Neu7/4). Macrophage numbers, however, were not changed significantly after LPS injection with or without BSCI treatment (Fig. 7 and Fig. S1). We noticed, however, a slight decrease in the number of macrophages in the control group treated with BSCI as compared to the control saline-treated group (22 ± 3 *versus* 30 ± 2). In contrast, there was a substantial neutrophil response 12 hrs after the systemic administration of LPS manifested by a significant sixfold increase in myometrial and twofold increase in decidual neutrophils as compared to saline group (*P* < 0.05, Fig. 7). Importantly, pre-treatment of pregnant mice with BSCI 24 hrs prior to the LPS injection significantly inhibited neutrophil accumulation in myometrium (34.3 ± 1.3 *versus* 25.5 ± 2.5, *P* < 0.05) but not in decidua (19.5 ± 4.3 *versus* 18 ± 3.3). BSCI alone, however, did not influence the number of neutrophils measured in reproductive tissues, myometrium and decidua as compared to the control vehicle-treated animals. We cannot exclude the possibility that some of the positive cells could represent monocytes as they can faintly express both markers. It was previously reported that Neu7/4 can recognize monocytes along with neutrophils and that F4/80 has low expression on monocytes [33].

## Discussion

Uterine tissues from preterm deliveries (with and without infection) show a correlation between cytokine levels and the leucocyte infiltration, suggesting a direct link between the host response to infection and the onset of PTB [13,34]. However, to date tocolytic therapies targeting specific chemokines in the inflammatory pathway have had little effect because of the redundancy of the system which suggest the necessity to block multiple pathways that trigger the onset of labour at the same time. The aim of this study was to examine whether blocking the signalling of multiple chemokines (through administration of BSCI) might suppress the inflammatory reaction associated with PTL and therefore decrease the rate of infectious PTB. We show that systemic LPS administration to pregnant CD-1 mice resulted in PTB within 24 hrs and was associated with a rapid increase in maternal plasma cytokines and an acute inflammatory response in uterine tissues leading to a sixfold increase in myometrial neutrophil/monocyte numbers. The application of BSCI compound ‘BN83470’: (*i*) significantly reduced the incidence of LPS-mediated PTB (from 100% to 64%) and delayed the onset of PTB (from 20.1 to 45.2 hrs); (*ii*) attenuated TNF-α, IL-6 and Csf2 protein levels in maternal plasma (*P* < 0.05); (*iii*) inhibited myometrial pro-inflammatory cytokines and chemokine gene expression (*IL-1*β, *IL-6*, *IL-12*, *Cxcl1*, *Cxcl2*, *Ccl2*, *Ccl4*, *Csf2*; *P* < 0.05); and 4) significantly decreased the number of peripheral leucocytes infiltrating myometrium.

Chemokines and their receptors are involved in the development of tissue inflammation. It has been suggested that broad spectrum blockage of chemokine function using a new class of anti-inflammatory agents with the ability to inhibit the trafficking of leucocytes could be effective in preventing the development of inflammatory diseases *in vivo*. Studies have shown that targeting inflammatory pathways is effective in alleviating symptoms in a number of disease models such as atherosclerosis [29], ischaemia [35,36], lung disease [30], surgical adhesions [37], endometriosis [38] and pulmonary disease [39], inhibited HIV replication [31]; though multiple side effects associated with this therapy have been reported [28]. Further investigation led to the development of 3-acylaminolactams [40], which are small anti-inflammatory molecules with increased pharmacological potencies *in vitro* and high anti-inflammatory activity *in vivo* [41]. These new BSCIs neither block chemokines binding to their receptors nor block the receptors themselves; instead they bind to the type-2 somatostatin receptor on the cell surface previously unconnected with chemokine signalling [42]. The presence of somatostatin and its receptors was detected in cells mediating inflammation and immune response such as lymphocytes, monocytes and granulocytes [43]. BSCI possibly exerts its inhibitory actions by converting chemokine receptors into inactive docking sites that bind to chemokines without eliciting downstream signalling/effects [28]. Interestingly, BSCI does not affect migration induced by non-chemokine stimuli and have minimal side effects [28].

Plasma levels of TNF-α measured 2 hrs after LPS injection is a rapid *in vivo* screen of antibiotics anti-inflammatory activity widely adopted by pharmaceutical companies [41]. It was previously reported by Funxional Therapeutics Ltd that TNF-α levels were decreased in plasma of BSCI-treated mice in an *in vivo* inflammatory model of murine sub-lethal endotoxaemia [41]. In our study, we showed that not only TNF-α, but also IL-6, Csf2 (*P* < 0.001) and IL-1b (*P* = 0.07) protein expression was inhibited by BSCI in maternal plasma after low-dose systemic LPS administration. Similarly, natural somatostatin (SS-14) has been shown to be capable of modifying TNF-α secretion in a rat model of septic shock induced by bacterial LPS [44]. Cortistatin, novel somatostatin-related cyclic neuropeptide, down-regulated the production of inflammatory mediators by LPS-activated macrophages [45]. This therapeutic effect on lethal endotoxaemia was mediated by decreasing the local and systemic levels of a wide spectrum of inflammatory mediators and acute phase proteins [45]. In addition, octreotide (a synthetic somatostatin analogue) reduced the severity of sepsis-induced oxidative injury in rat uterine and ovarian tissues by diminishing neutrophil infiltration [46]. This corresponds well with our data indicating that BN83470 can protect reproductive tissues (especially the myometrium) from inflammatory injury by suppressing the infiltration of neutrophils, an important source of oxygen free radicals.

Our data provide the first evidence of BSCIs as new immunomodulatory factors with the capacity to prevent PTB in mice by deactivating the systemic inflammatory response in the maternal blood and in reproductive tissues. We appreciate that our animal model of PTB does not mimic all cases of human PTB, however we opted to use this model of systemic illness for the ‘proof of principle’ study of BSCI effect. The therapeutic effect of BSCI (BN83470) was mediated by decreasing the local and systemic levels of pro-inflammatory cytokines and chemokines in pregnant mice. These results again highlight the role of cytokines in the process of labour initiation and also confirm the importance of the communication between maternal reproductive tissues and immune system as attenuated inflammation in uterine tissues can reduce the incidence of infection-mediated PTB.

Importantly, three of the genes responsible for attracting neutrophils and enabling their differentiation (*Cxcl1*, *Cxcl2* and *Csf2*) were down-regulated by BSCI in the placenta of animals challenged with LPS, which potentially prevented foetal exposure and inhibited foetal inflammatory response. In contrast to human, mouse placental barrier is formed by two layers of syncytiotrophoblasts. The studied drug compound needs to cross these layers by active or passive transport or by diffusion which is unlikely because of the chemical structure of BSCI (N-substituted-aminocaprolactam). Therefore, we speculate that this drug is not able to cross the placenta, as we could not detect any effect of BSCI on the chemokine profile in the amniotic fluid of animals systemically challenged with LPS. Our current data suggest that BSCI therapy itself (multiple daily injections of the drug) did not influence foetal viability or placental weights when administered daily during the last trimester of pregnancy in mice. Interestingly, the birth weights were significantly higher in the animal group treated with BSCI starting from GD14 as compared to mice that were treated with BSCI and induced with LPS at GD15. This suggests that BSCI may not exhibit any significant foetotoxicity. However, extensive studies focusing on the careful monitoring of foetal development and well-being, on the effect on BSCI on the foetal immune response, on the analysis of the maternal blood and urine are necessary to test potential side effects of BSCI administered during pregnancy.

In summary, while our results suggest that targeting the activation of peripheral maternal immune cells using BSCIs is an effective means of preventing PTB induced by systemic infection, many questions remain. For example, will BSCI be effective in situations where labour has already been initiated or should it be only used as a prophylactic therapy; are these drugs equally effective in different models of PTB (systemic, intrauterine or cervical routes of infection)? These very important questions could be resolved in future by using different animal models of PTB and different timing of BSCI administration. In addition, the availability of a new generation of orally active BSCIs (such as FX125L from Funxional Therapeutics Ltd) with considerably superior pharmaceutical properties, including pharmacokinetics, potency, safety and toxicology offers the potential for greater therapeutic efficacy. The effectiveness of FX125L has been demonstrated in a wide range of animal models of disease *in vivo*, including allergic asthma, rheumatoid arthritis, diabetic nephropathy and surgical adhesions. Based on our current understanding of the importance of infection/inflammation in the aetiology of PTB, we would anticipate that FX125L represents a therapeutic class of compounds that may be effective in preventing PTB in humans.

## Supporting information

**Figure S1** *In situ* localization of leucocytes in mouse uterus after treatment with either LPS or saline ± BSCI. Immunohistochemical examination was performed on sections of uterus from gestational days 15.5 collected 12 hrs after LPS and/or BSCI administration. Neutrophils were identified using anti-Neu7/4 antibody and macrophages- using anti-F4/80 antibody. Representative photomicrographs of (A) neutrophils (Neu7/4) and (B) macrophages (F4/80) in the myometrium (M) and decidua (D) are shown. Leucocytes are stained dark brown in paraffin-embedded tissue sections that were counterstained a blue with Haematoxylin. The negative controls were performed on GD15 uterine tissue samples: Control 1 represents the lack of immunostaining after incubation of myometrial tissue with non-specific rat IgG and Control 2 shows staining with anti-rat secondary antibodies in the absence of primary antibody. Magnification = 100×; scale = 200 μm.
