# Supplementary material for: Inhibition of infection-mediated preterm birth by administration of broad spectrum chemokine inhibitor in mice
Source: J Cell Mol Med. 2014 Jun 4;18(9):1816–29. doi: 10.1111/jcmm.12307 (PMC4196657; doi:10.1111/jcmm.12307)
Supplement: Supplementary file 2 — Figure S1 In situ localization of leucocytes in mouse uterus after treatment with either LPS or saline ± BSCI. Immunohistochemical examination was performed on sections of uterus from gestational days 15.5 collected 12 hrs after LPS and/or BSCI administration. Neutrophils were identified using anti-Neu7/4 antibody and macrophages- using anti-F4/80 antibody. Representative photomicrographs of (A) neutrophils (Neu7/4) and (B) macrophages (F4/80) in the myometrium (M) and decidua (D) are shown. Leucocytes are stained dark brown in paraffin-embedded tissue sections that were counterstained a blue with Haematoxylin. The negative controls were performed on GD15 uterine tissue samples: Control 1 represents the lack of immunostaining after incubation of myometrial tissue with non-specific rat IgG and Control 2 shows staining with anti-rat secondary antibodies in the absence of primary antibody. Magnification = 100×; scale = 200 μm. [file jcmm0018-1816-SD2.docx]

**Supplemental Figure 1.** ***In situ* localization of leukocytes in mouse uterus after treatment with either LPS or Saline ±BSCI.** Immunohistochemical examination was performed on sections of uterus from gestational days 15.5 collected 12 hours after LPS and/or BSCI administration. Neutrophils were identified using anti-Neu7/4 antibody and macrophages - using anti-F4/80 antibody. Representative photomicrographs of (A) neutrophils (Neu7/4) and (B) macrophages (F4/80) in the myometrium (M) and decidua (D) are shown. Leukocytes are stained dark brown in paraffin-embedded tissue sections that were counterstained a blue with Hematoxylin. The negative controls were performed on GD15 uterine tissue samples: Control 1 represents the lack of immunostaining after incubation of myometrial tissue with non-specific rat IgG and Control 2 shows staining with anti-rat secondary antibodies in the absence of primary antibody. Magnification = 100x; scale =200 μm.
